# Nicotinamide Protects against Ethanol-Induced Apoptotic Neurodegeneration in the Developing Mouse Brain

**DOI:** 10.1371/journal.pmed.0030101

**Published:** 2006-02-21

**Authors:** Alessandro Ieraci, Daniel G Herrera

**Affiliations:** 1Department of Psychiatry, Weill Medical College of Cornell University, New York, New York, United States of America; National Institute of Child Health and Human DevelopmentUnited States of America

## Abstract

**Background:**

Exposure to alcohol during brain development may cause a neurological syndrome called fetal alcohol syndrome (FAS). Ethanol induces apoptotic neuronal death at specific developmental stages, particularly during the brain-growth spurt, which occurs from the beginning of third trimester of gestation and continues for several years after birth in humans, whilst occuring in the first two postnatal weeks in mice. Administration of a single dose of ethanol in 7-d postnatal (P7) mice triggers activation of caspase-3 and widespread apoptotic neuronal death in the forebrain, providing a possible explanation for the microencephaly observed in human FAS. The present study was aimed at determining whether nicotinamide may prevent ethanol-induced neurodegeneration.

**Methods and Findings:**

P7 mice were treated with a single dose of ethanol (5g/kg), and nicotinamide was administered from 0 h to 8 h after ethanol exposure. The effects of nicotinamide on ethanol-induced activation of caspase-3 and release of cytochrome-c from the mitochondria were analyzed by Western blot ( *n* = 4–7/group). Density of Fluoro-Jade B–positive cells and NeuN-positive cells was determined in the cingulated cortex, CA1 region of the hippocampus, and lateral dorsal nucleus of the thalamus ( *n* = 5–6/group). Open field, plus maze, and fear conditioning tests were used to study the behavior in adult mice ( *n* = 31–34/group). Nicotinamide reduced the activation of caspase-3 (85.14 ± 4.1%) and the release of cytochrome-c (80.78 ± 4.39%) in postnatal mouse forebrain, too. Nicotinamide prevented also the ethanol-induced increase of apoptosis. We demonstrated that ethanol-exposed mice showed impaired performance in the fear conditioning test and increased activity in the open field and in the plus maze. Administration of nicotinamide prevented all these behavioral abnormalities in ethanol-exposed mice.

**Conclusions:**

Our findings indicate that nicotinamide can prevent some of the deleterious effects of ethanol on the developing mouse brain when given shortly after ethanol exposure. These results suggest that nicotinamide, which has been used in humans for the treatment of diabetes and bullous pemphigoid, may hold promise as a preventive therapy of FAS.

## Abstract 2

Nicotinamide, a drug used routinely by human patients with a variety of conditions, is able to prevent some of the neurotoxic and behavioral effects of ethanol exposure in a mouse model of fetal alcohol syndrome.

## Introduction

Ethanol exposure during brain development can provoke neurodevelopmental defects referred to as fetal alcohol effects (FAE) or fetal alcohol syndrome (FAS), depending on their severity [ 1], with an array of neurological disorders including hyperactivity, learning and memory deficits, mental retardation, psychosis, depression, and schizophrenia [ 2, 3]. A number of mechanisms have been proposed to contribute to ethanol neurotoxicity: oxidative stress, induction of apoptosis, excitotoxicity, disruption of cell–cell interaction, and interference with the activity of growth factors [ 4]. Several lines of evidence point out that consuming many drinks per occasion (i.e., binge drinking) is particularly harmful to the developing brain [ 5]. Ethanol may damage the developing brain by affecting neurogenesis, migration, or survival of cells [ 6, 7]. Neurons are more susceptible to ethanol-induced apoptotic cell death during synaptogenesis, also known as the brain growth spurt [ 8, 9], which in humans takes place during the third trimester of pregnancy, continuing for several years after birth [ 10]. Epidemiological studies indicate that FAS is the major non-genetic cause of mental retardation in the Western world [ 11]. Despite attempts to increase awareness of FAS, consumption of alcohol during pregnancy, especially binge drinking, has increased in recent years, and currently there are no effective treatments to prevent or revert FAS after ethanol exposure.

Studies of alcohol exposure in developmental animal models are excellent tools to identify possible mechanisms and interventions that may prevent or attenuate ethanol's effect on the developing brain. A single heavy episode of ethanol exposure (binge-like drinking) in 7-d postnatal (P7) mice, a period of brain development that is comparable to the human third trimester, triggers widespread neurodegeneration, resulting in the loss of millions of neurons [ 12]. Transplacental ethanol exposure in guinea pigs during synaptogenesis (a prenatal phenomenon in this species) promotes a similarly intense apoptotic response in the fetal brain [ 13]. Neurodegeneration could provide a likely explanation for the reduced brain mass and neurobehavioral disturbances observed in human FAS. The cell death produced by ethanol in mice appears to be associated with activation of caspase-3, an executioner protease that is activated during apoptotic cell death [ 14, 15].

Nicotinamide, an amide of vitamin B3, is the precursor for the coenzyme β-nicotinamide adenine dinucleotide (NAD+) and is considered to be necessary for cellular function and metabolism. Recently, interest in nicotinamide has shifted from its role as a nutrient to that as a novel neuroprotective agent [ 16]. Treatment with nicotinamide enhances neuronal survival during a variety of insults such as free radical exposure and oxidative stress [ 17, 18], and in animals improves neurological outcome and reduces infarct volume in transient and permanent focal ischemia in vivo [ 19– 21]. The mechanism of nicotinamide-mediated neuroprotection in vitro may be in part due to inhibition of caspase-3 and release of cytochrome-c from mitochondria during oxygen–glucose deprivation [ 22, 23].

Based on these observations, we investigate whether nicotinamide is able to prevent the neurotoxic effects of ethanol exposure in the postnatal brain of mice. Furthermore, we analyze whether inhibition of ethanol-induced apoptotic neuronal death is sufficient to prevent behavioral impairment in adult mice.

## Methods

### Animals

P7 CD1 mice were injected subcutaneously with 20% ethanol in saline solution delivering 5g/kg body weight, and sacrificed at several time points. Nicotinamide and 1-methyl-nicotinamide (Sigma, St. Louis, Missouri, United States) were dissolved in saline solution (50mg/ml) and were administered subcutaneously at different doses (0.25, 0.5, and 1mg/g) and time points (0, 2, 4, or 8 h) after ethanol injection. Saline injections of equal volume were used as controls. In each litter, animals were equally distributed into the different treatment groups (see Table S1). Animal care was provided according to the guidelines of Weill Medical College of Cornell University.

### Western Blot Analysis

Animals were killed at 2, 4, 8, 12, and 24 h after ethanol administration (see Table S1). Brains were rapidly dissected out, and cerebellum, brain stem, and olfactory bulb were carefully removed and tissue was frozen in dry ice. For each treatment group, four to seven pups from 14 different litters were analyzed.

### Caspase-3 Western Blot

Caspase-3 analysis was done as previously described with some modifications [ 15]. Forebrains were divided in half and lysed in boiling SDS buffer (250 mM tris-HCl [pH 6.8], 2.5% SDS) followed by brief sonication and further centrifuged at 14,000 g for 20 min. Supernatant was collected and stored at −80 °C until further analysis.

### Cytochrome-C Western Blot

In order to analyze the release of cytochrome-c from the mitochondria into the cytoplasm, fresh tissue was prepared as previously described [ 24]. The forebrain was dissected in half, each sample cut into small pieces, and resuspended in ice cold buffer (250 mM sucrose, 20 mM HEPES, 10 mM KCl, 1.5 mM MgCl _2_, 1 mM EDTA, 1 mM EGTA, 1 mM DTT, 1 mM PMSF, 1 mM aprotinin, 1mM leupeptin) for 10 min. Tissue was homogenized by 30 strokes with a glass Dounce using a loose pestle. Intact cell and nuclei were centrifuged for 10 min at 750 g. The supernatant was further centrifuged at 14,000 g for 20 min, and the resultant supernatant was considered as cytosolic fraction and stored at −80 °C until further analysis.

Material coming from different brains was never pooled. Protein concentration was determined with a DC Protein Assay Kit (Bio-Rad, Hercules, California, United States). Equal amounts of protein (50 μg) were boiled for 5 min in SDS loading buffer (50 mM Tris-Cl [pH 6.8], 2% SDS, 2% β-Mercaptoethanol, 0.1% bromophenol blue, 10% glycerol); resolved by 12%–15% SDS-PAGE gel, and transferred to PVDF membranes (Immobilon P, Millipore, Bedford, Massachusetts, United States) in a BioRad wet transfer unit. After transfer was completed, membranes were stained with Ponceau Red (Sigma) to verify loading, sample integrity, and protein separation. Membranes were blocked with 5% nonfat milk in TBS (10 mM Tris [pH 7.4]; 150 mM NaCl, 0.1% Tween 20) for 1 h, and then incubated overnight at 4 °C with primary antibody. The following antibodies were used: anti-active caspase-3 antibody (1:1,000; Cell Signalling, Beverly, Massachusetts, United States), anti-cytochrome-c antibody (1:1,000; Pharmingen, San Diego, California, United States), anti-α-tubulin antibody (1:40,000; Sigma). Washed membranes were incubated with the appropriate horseradish peroxidase-conjugated secondary antibody (1:4000, Calbiochem, San Diego, California, United States) 1 h at room temperature, and immunoblotted proteins were detected by an enhanced chemiluminiscent method (Pierce, Rockford, Illinois, United States). For densitometric analysis, immunoreactive bands were scanned and intensity quantitated using NIH Image software (Scion, Frederick, Maryland, United States).

### Ethanol Level Quantification

Blood and brain samples were collected at 4, 8, 12, and 24 h after ethanol administration (see Table S1). Mice from eight litters were sampled ( *n* = 4 for each treatment group for each time point). Blood was centrifuged at 3000 g for 20 min and plasma stored at −20 °C until assayed. Brains were divided in half and lysed in 600 μl of cold EB buffer (1% Triton X-100, 10 mM Tris-HCL [pH7.5], 150mM NaCl, 5 mM EDTA, 10% Glycerol), centrifuged at 14,000 g for 20 min at 4 °C, supernatant was collected and stored at −80 °C until assayed. Aliquots were taken to measure protein concentration. 10 μl of plasma and supernatant were used to determined ethanol level using the Ethanol Assay kit (DCL, Charlottetown, Prince Edward Island, Canada) in accordance with the manufacturer's recommendations. All samples were run in duplicate and averaged.

### Morphological Analysis

Animals were sacrificed 24 h (Fluoro-Jade B) or 14 d (NeuN [neuronal nuclear protein]) after ethanol treatment (see Table S1). Five to six mice from a total of four different litters were analyzed for each treatment group. After intracardial perfusion with 4% of paraformaldehyde, brains were removed and post-fixed overnight at 4 °C. Brains were cut into 40-μm–thick transverse sections on a vibratome.

### Fluoro-Jade–B Staining

Fluoro-Jade–B staining was performed as previously described [ 25]. Briefly, sections were mounted on slides and then air dried over night. The slides were immersed in a solution of 1% sodium hydroxide and 80% ethanol for 5 min, then in 70% alcohol for 2 min followed by 2 min in distilled water. Slides were transferred in 0.06% potassium permanganate solution for 10 min. After rinsed in water, slides were immersed in a solution of 0.1% acetic acid and 0.0004% Fluoro-Jade B (Calbiochem, San Diego, California, United States) for 20 min. Slides were washed 3× in distillated water, allowed to dry at 55 °C for 10 min, and mounted with Krystalon (EMD Chemicals, Gibbstown, New Jersey, United States).

### NeuN Immunohistochemistry

For NeuN immunohistochemistry, floating sections were incubated for 20 min in 3% H _2_O _2_. After rinsing in PBS, sections were incubated 30 min in blocking solution (PBS, 10% goat serum, 0.3% Triton-X100). Sections were then incubated with an anti-NeuN antibody (1:500; Chemicon, Temecular, California, United States) overnight at 4 °C. Sections were rinsed and incubated 1 h at room temperature with biotinylated anti-mouse (1:200; Chemicon), followed by the peroxidase-based Vectstain ABC system (Vector Labs, Burlingame, California, United States) using diaminobenzidine (Sigma) as the chromogen. Staining with NeuN is markedly superior to that produced by the Nissl method: neurons are stained with a greater intensity and with more defined contours (personal observation and [ 26]). Counting of neuronal populations with NeuN immunostaining is not only comparable to that done with Nissl but may also reduce errors when counting small neurons [ 26].

### Cell Counting

Cells were counted one section out of every six (240 μm apart from each other) throughout the brain by using a Nikon Eclipse E600 with a Nikon 40× objective. Two to four sections per brain for the LDN (lateral dorsal nucleus) of thalamus, and six to ten sections per brain for the CA1 and the cingulate cortex were counted in five to six animals of each group. Cells were counted in both hemispheres. Contours of Cingulate CX (Bregma from 1.34 to −0.82), CA1 (dorsal part Bregma from −1.22 to −1.84; ventral part Bregma from −3.52 to −.88) and LDN (Bregma from −1.06 to −1.46) [ 27] were traced using a personal computer, and the area was calculated with Stereo Investigator software (MBF Bioscience, Williston, Vermont, United States). The section thickness was measured at different random locations, and the range was: 8–12 μm for Fluoro-Jade B and 18–22 μm for NeuN stain. The relative area densities (counts/mm ^2^) of Fluoro-Jade B–positive cell profiles were estimated using the fractionator probe and systematic random sampling (MBF Bioscience), and counting was done between 2–5 d after staining the sections for Fluoro-Jade B. There was no significant fading of fluorescence when randomly selected sections were recounted blindly 10 d later, agreeing with previously published data [ 25]. Pilot studies revealed no consistent differences in profile size between control and treated animals. The density of NeuN+ cells (counts/mm ^3^) was estimated with the optical dissector method, which has been described previously [ 28– 30]. The results were normalized and expressed as percentage of control. Briefly, the number of neurons (Q) was counted in a dissector volume (V _dis_) defined by the product of a counting frame and the distance between two dissectors planes ( *h*). The density of cells (N _v_) was determined by dividing the total number of counting cells (Q) by the total volume in which cells were counted (V _dis_). Twenty-five to 30 different fields were randomly selected for each region in each section analyzed and a total of 200–400 cells/region/brain were counted with a CE of 0.07–0.05. The dimension of the counting frame was 50 × 50 μm with a dissector depth *(h)* of 12 μm for the cingulate cortex and the LDN of thalamus, while for the CA1 region it was 20 × 20 μm with a dissector depth of 8 μm. A 2-μm guard space from the top was used. Counting was conducted in a blinded manner.

### Behavioral Tests

P7 mice received subcutaneous injection of saline or ethanol, and nicotinamide was administrated 2 h later. The different treatments were distributed across sexes and litters as evenly as possible. Animals remained with the lactating dam until P21, at which time they were separated by sex and housed under a 12:12 h light/dark cycle in a temperature- and humidity-controlled animal facility. Behavioral tests were started on P60 and the total time necessary to run all tests was 5–6 wk. Thirty-one to 34 animals (from a total of 16 different litters) were sampled for each treatment group (see Table S1). All the experiments were conducted in a blind manner. Animals were separated by treatment and four to five animals were housed per cage. A person who did not know how the mice have been treated coded each cage. At the end of the entire behavioral test, the codes were opened. Mice were tested in open field, plus maze, and fear conditioning tests, tests widely used to study behavioral changes in animal expose to ethanol in the perinatal period [ 31– 34].

### Open Field

The open field test was performed as described previously [ 33, 35]. Mice were placed in a 50 × 50 cm square chamber for 5 min. The floor was divided into nine squares. The test was conducted in the dark. Each mouse was place in the center of the box. Each session was videotaped. Horizontal (lines crossed) and vertical (rearing) activities were recorded.

### Elevated Plus Maze

Elevated plus maze was performed as described previously [ 33, 36]. The apparatus consisted of two open arms (30 × 5 cm), two closed arms (30 × 5 cm with 15 cm high black wall), and a connecting central platform (5 × 5 cm). Each mouse was placed in the center facing one open arm and then allowed to explore the maze for 5 min. The test was conducted in the dark light. Animals were videotaped and scored for total entries into all arms, percent of total entries into the open arm, and percent of total time spent in the open arms.

### Fear Conditioning

The fear conditioning test was performed as described previously [ 31, 37]. On day 1 of training, mice were placed individually in the conditioning chamber (chamber A) (Coulbourn Instruments, Allentown, Pennsylvania, United States). After 2 min of habituation, three tone-shock pairs were given at 1-min intervals. Each tone-shock pair consisted of a 20-s acoustic white noise tone (CS) (70db, 2.9KHz), followed by a 1-s foot shock (US). One minute after the third shock, mice were returned to their home cage. On day 2, mice were placed in the same environment (contextual conditioning), and the total freezing (cessation of all movement other than respiration) time was measured for 5 min. On day 3, mice were placed in a different chamber (chamber B) and after 120 s three separate CS of 20 s were delivered at 1 min intervals (cued conditioning). Freezing was scored and expressed as the percentage of time spent freezing. To exclude the possibility that differences in the time freezing were due to an altered activity, the basal level of freezing in the animals was scored for 2 min the first day before the training (pre-testing) and the third day before the presentation of the tone (pre-tone). Some of the animals were not scored in the pre-training and in the pre-tone analysis because the videotape was not available.

### Data Analysis

Statistical analyses were performed with STAT VIEW software (SAS Institute, Cary, North Carolina, United States). Data are presented as mean ± standard deviation (SD). Statistical analyses were made using analysis of variance (ANOVA), and Bonferroni's procedure was used for multiple comparison analysis.

## Results

### Nicotinamide Inhibits Ethanol-Induced Apoptotic Pathway

P7 mice were injected subcutaneously with a 20% solution of ethanol in normal saline delivering 5 g/kg, and sacrificed at different time points. This dose maintained a blood ethanol concentration above 200 mg/dl for several hours, which is the minimum level needed for triggering neurodegeneration consistently [ 12, 14]. This blood ethanol level is in the range that a human fetus might be exposed to after maternal ingestion of a moderate to heavy dose of ethanol [ 38]. A clear activation of caspase-3 was observed 4 h after ethanol administration; it peaked at 12 h and was still evident 24 h after ethanol treatment ( Figure S1A).

**Figure 1:**
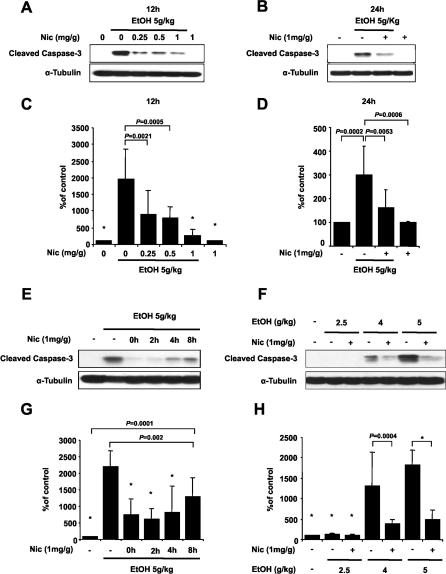
Nicotinamide Inhibits Ethanol-Induced Caspase-3 Activation (A–D) P7 mice were injected with ethanol and received nicotinamide 2 h later. (A and B) Level of cleaved caspase-3 was analyzed by Western blot at 12 h (A) and 24 h (B) after ethanol administration with different doses of nicotinamide. (C and D) Densitometric quantification of cleaved caspase-3 at 12 h (ANOVA *F _5,27_* = 10.989; *p* < 0.0001; *n* = 5–6 each treatment group) (C) and 24 h (ANOVA *F _3,18_* = 8.851; *p* = 0.0008; *n* = 6 each treatment group) (D). (E and G) Nicotinamide was administered to P7 mice at different time points after ethanol exposure. Western blot analysis of caspase-3 activation 12 h after ethanol injection (E) and densitometric quantification (ANOVA *F _5,33_* = 15.061; *p <* 0.0001; *n* = 6–7 each treatment group) (G). (F and H) Different doses of ethanol were administered to P7 mice and nicotinamide was injected 2 h later. Western blot analysis of caspase-3 activation 12 h after ethanol injection (F) and densitometric quantification (ANOVA *F _6,28_* = 18.183; *p <* 0.0001; *n* = 5 each treatment group) (H). Values are shown as mean ± SD. Bonferroni correction for multiple comparisons revealed a significant difference between the ethanol treatment group and all other groups. ∗ *p <* 0.0001.

In order to determine if nicotinamide can inhibit ethanol-induced caspase-3 activation, a series of different experiments was performed ( Figure 1). In a first set of experiments, increasing doses of nicotinamide were administered subcutaneously 2 h after ethanol exposure. 12 h after treatment, animals injected with ethanol alone showed a 15-fold to 20-fold increase of cleaved caspase-3 compared with a saline control ( Figure 1A and 1C; Table S2). Administration of nicotinamide reduced caspase-3 activation, and maximum inhibition was seen with a 1 mg/g dose ( Figure 1A and 1C; Table S2). To rule out the possibility that nicotinamide might only delay caspase-3 activation, we evaluated the effects of nicotinamide on caspase-3 activation 24 h after ethanol administration. Cleaved caspase-3 was increased 3-fold–4-fold in ethanol-treated mice compared with controls ( Figure 1B and 1D; Table S3). However, one single injection of nicotinamide 2 h after ethanol administration was sufficient to reduce ethanol-induced caspase-3 activation by approximately 50% after 24 h ( Figure 1B and 1D; Table S3).

In a second set of experiments, animals received nicotinamide (1 mg/g) at 0, 2, 4, or 8 h after ethanol administration. Activation of caspase-3 was assessed 12 h after ethanol exposure. We found that nicotinamide treatment prevented ethanol-induced activation of caspase-3 at all time points tested, and the strongest effect was observed when nicotinamide was administered between 0 h and 2 h after ethanol exposure ( Figure 1E and 1G; Table S4).

To verify that nicotinamide was also able to prevent the ethanol-induced activation of caspase-3 at a lower ethanol concentration, mice were exposed to 2.5, 4, or 5g/kg of ethanol, and nicotinamide was injected 2 h later. A dose of 2.5 g/kg was not enough to consistently detect caspase-3 activation 12 h after ethanol treatment in the whole forebrain ( Figure 1F and 1H). Nicotinamide administration prevented ethanol-induced activation of caspase-3 at 4 or 5g/kg of ethanol exposure ( Figure 1F and 1H; Table S5).

Finally, to test the specificity of actions of nicotinamide, we administrated 1-methyl-nicotinamide, an analogue of nicotinamide, 2 h after ethanol exposure. This analogue is not converted into NAD+, and its administration did not prevent ethanol-induced caspase-3 activation (Figure S2B and S2C; Table S6).

Based on these findings, ethanol was administered at 5 g/kg and nicotinamide at 1 mg/g, 2 h after ethanol treatment in the rest of the experiments.

**Figure 2:**
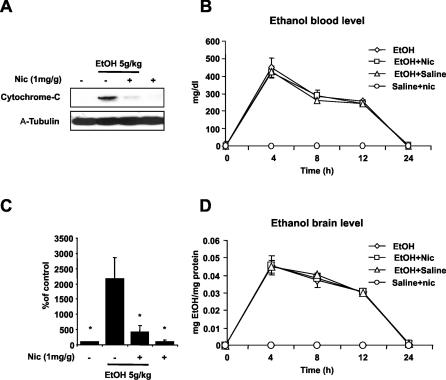
Nicotinamide Blocks Release of Cytochrome-C from Mitochondria (A) Western blot analysis of cytochrome-c release in the cytosolic fraction 12 h after ethanol administration. (C) Densitometric quantification of cytochrome-c release (ANOVA *F _3,20_* = 43.546 *p <* 0.0001; *n* = 6 each treatment group). Values are shown as mean ± SD. Bonferroni correction for multiple comparison revealed a significant difference between ethanol treatment group and all other groups. ∗ *p <* 0.0001. (B and D) Nicotinamide (1mg/g) does not alter ethanol absorption or excretion. Ethanol levels in blood (B) and brain (D) were measured at different time points after 5g/kg of ethanol treatment in P7 mice. Nicotinamide was administered 2 h after ethanol exposure. No significant differences were seen between the different treatments ( *n* = 4 each treatment group).

Release of cytochrome-c into the cytoplasm induces the formation of apoptosome, which in turn activates caspase-3 [ 14, 23]. Recently, it has been shown that ethanol-induced neuronal death is Bax-dependent and involves the release of cytochrome-c from the mitochondria [ 39]. Since nicotinamide can block the cytochrome-c release in vitro, we tested whether inhibition of cytochrome-c release might be the target of nicotinamide in vivo ( Figure 2). Figure 2A and 2C shows that in ethanol-treated pups there is a 20-fold increase of cytochrome-c release after 12 h compared with controls, and that administration of nicotinamide significantly reduced this release ( Figure 2A and 2C; Table S7).

### Nicotinamide Does Not Alter the Metabolism of Ethanol

Metabolism of ethanol through alcohol dehydrogenase requires NAD+ with production of acetaldehyde. Further oxidation of acetaldehyde is mediated predominantly by the mitochondrial aldehyde dehydrogenase, which also needs NAD+. Therefore, the rate-limiting factor in the metabolism of ethanol is the amount of NAD+. It has been shown that administration of nicotinamide increases the level of NAD+ [ 17]. To test if nicotinamide could modify the metabolism of ethanol, we measured alcohol level in the blood and in the brain at several time points after ethanol administration, with and without nicotinamide. Figure 2B and 2D shows that 1mg/g of nicotinamide did not change the pharmacokinetics of ethanol (as alcohol levels neither in the blood nor in the brain were affected) ( Figure 2B and 2D).

### Nicotinamide Inhibits Ethanol-Induced Neuronal Death

**Figure 3:**
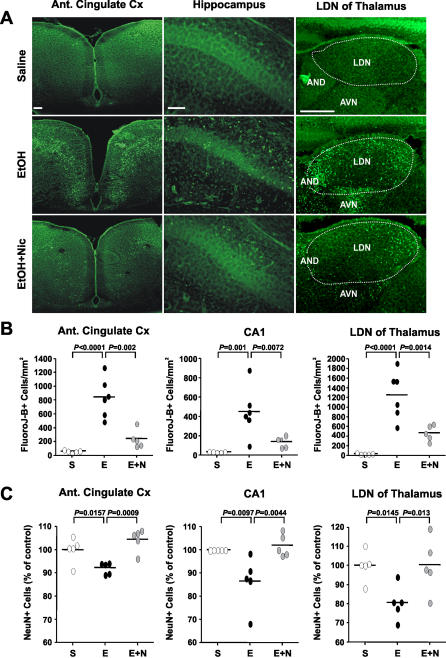
Nicotinamide Inhibits Ethanol-Induced Neurodegeneration (A) Fluoro-Jade B staining 24 h after ethanol treatments. (B) Quantification of Fluoro-Jade–B positive cells in the cingulate cortex (ANOVA *F _2,13_* = 25.541; *p <* 0.0001; *n* = 5–6 each treatment group), in the total CA1 region of hippocampus (ANOVA *F _2,13_* = 9.988; *p* = 0.0024; *n* = 5–6 each treatment group), and in the LDN of thalamus (ANOVA *F _2,13_* = 20.785; *p* < 0.0001; *n* = 5–6 each treatment group). (C) Total number of NeuN positive cells were counted 2 wk after ethanol administration in the cingulate cortex (ANOVA *F _2,12_* = 9.894; *p* = 0.0029; *n* = 5 each treatment group), in the CA1 region of hippocampus (ANOVA *F _2,12_* = 7.289; *p* = 0.0085; *n* = 5 each treatment group) and in the LDN of thalamus (ANOVA *F _2,12_* = 5.551; *p* = 0.0196; *n* = 5 each treatment group). Values are shown as mean ± SD. Bonferroni correction for multiple comparison revealed a significant difference between ethanol treatment group and all other groups. S, saline; E, ethanol; Etoh+Nic, ethanol + nicotinamide. Scale bars 200μm.

Following activation, caspase-3 is responsible for the cleavage of a broad spectrum of cellular targets, ultimately leading to cell death. In addition, other caspase-3 independent mechanisms may also be involved in apoptotic cell death. To determine whether inhibition of caspase-3 activation by nicotinamide is sufficient to prevent ethanol-induced cell death, brains were histologically analyzed for evidence of neurodegeneration. 24 h after ethanol treatment, Fluoro-Jade–B staining, predominantly a marker of neuronal injury [ 25], revealed neurodegeneration throughout the forebrain of ethanol-treated mice. In contrast, mice that received nicotinamide after ethanol administration showed a dramatic reduction in Fluoro-Jade–B labeling ( Figure 3A). Quantification of Fluoro-Jade–B positive cells in regions particularly sensitive to ethanol at this developmental stage (anterior cingulate cortex, CA1 of the hippocampus, and LDN of thalamus), confirmed that administration of nicotinamide reduced the number of neurons injured by ethanol exposure ( Figure 3B). Furthermore, using NeuN, a marker of mature neurons [ 26], we established that there was a significant decrease in the number of mature neurons in the anterior cingulate cortex (488,792 ± 11,828 versus 450,392 ± 5,548; *p* = 0.01) and in the LDN of the thalamus (255,588 ± 11,583 versus 206,022 ± 13,017; *p* = 0.004) 14 d after ethanol exposure. In the hippocampus, while there was only a trend in the dorsal CA1 region (641,056 ± 38,388 versus 570,796 ± 26,311; *p* = 0.08), a significant statistical difference was present in the ventral region (707,552 ± 47,241 versus 556,756 ± 21,322; *p* = 0.02). Nicotinamide treatment prevented the ethanol-induced decrease of the number of neurons in all of these regions ( Figure 3C).

### Nicotinamide Prevents Ethanol-Induced Behavioral Impairment in Adult Mice

Rodents exposed to ethanol during early development show several behavioral and cognitive impairments as adults, and these impairments appear to correlate with ethanol-induced neuronal loss [ 33, 40– 42]. We have shown here that nicotinamide inhibits the decrease in the number of neurons following ethanol exposure during early postnatal development. To examine if this effect on neuronal death could prevent behavioral deficits, we conducted a series of behavioral tests on adult mice that had been exposed to ethanol during early development. Animals were exposed to either saline, ethanol, ethanol/nicotinamide, or saline/nicotinamide at P7. Mice were tested starting at 2 mo of age, and all behavioral tests were conducted blind to the treatment. First, we assessed spontaneous locomotor activity by placing the mice in the open field box for 5 min. Ethanol-treated mice showed increases in the number of lines crossed (horizontal activity) but not in the number of rears (vertical activity) ( Table 1 and unpublished data). Administration of 1 mg/g nicotinamide alone did not influence the spontaneous activity of the mice, compared with saline-treated mice ( Table 1). However when nicotinamide was administrated after ethanol in P7 mice, it completely prevented the increase of activity exhibited in adult ethanol-treated mice ( Table 1).

**Table 1:**
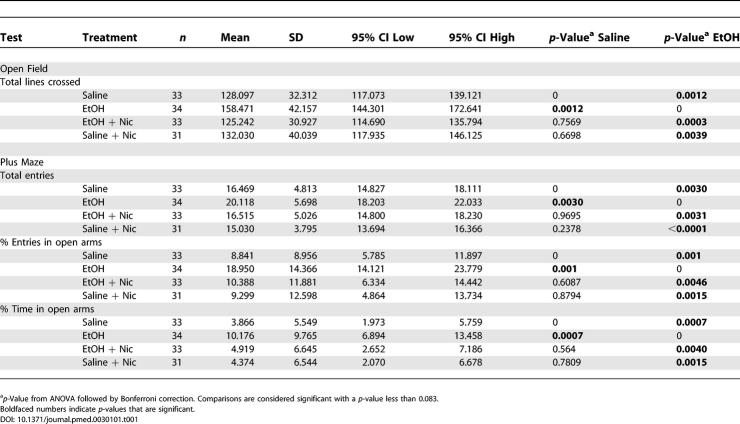
Nicotinamide Reverts Ethanol-Induced Hyperactivity in Adult Mice

The mice were then tested in the elevated plus maze. This task relies on the conflict between a mouse's natural aversion to open space and its exploratory behavior. Entries into the open arms are indicative of reduced anxiety or fear, while the total entries into all the arms correlate with activity level [ 35]. Ethanol-treated mice showed an increase in the total number of entries into both open and closed arms, indicating increased activity ( Table 1). Furthermore, ethanol-treated mice made more entries into the open arms and also stayed there for longer periods of time ( Table 1). In contrast, ethanol-treated mice that received nicotinamide did not show any differences when compared with saline-treated mice ( Table 1).

Finally, to test hippocampal learning and memory we used a fear-conditioning paradigm. In this test, mice learn to associate a context (experimental chamber) or cue (tone) with a foot shock. Contextual fear conditioning is hippocampus-dependent, while cued fear conditioning is hippocampus-independent. Prior to presentation of the first shock/tone pairing, all the mice showed a low level of freezing, and ANOVA analysis did not show any difference between the different groups (F _3,65_ = 2.271; *p* = 0.0885; *n* = 16–18 each treatment group) ( Table 2). 24 h after training, ethanol-treated mice showed significant impairment in contextual fear conditioning, displaying a significantly decreased level of freezing. Nicotinamide administration completely prevented the performance impairment induced by ethanol ( Table 2). Furthermore, 48 h after training, the same mice were tested with the conditioned stimulus (tone) in a novel chamber. All groups displayed similar and statistically indistinguishable freezing responses in both the new context before (pre-tone: ANOVA *F _3,122_* = 1.074; *p* = 0.3626; *n* = 31–33 each treatment group) and during the presentation of tone cue (tone: ANOVA *F _3,128_* = 1.312; *p* = 0.2733; *n* = 31–34 each treatment group) ( Table 2). These results suggest that exposure to ethanol, in the postnatal period, affects hippocampal-dependent memory in adult mice.

**Table 2:**
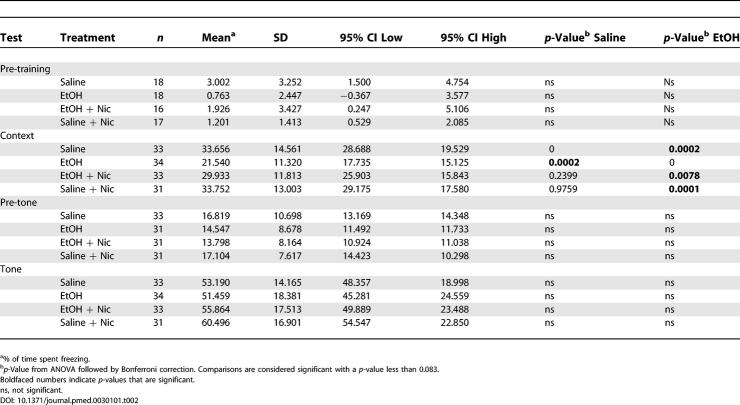
Nicotinamide Prevents Ethanol-Induced Impairment Performance in the Contextual Fear-Conditioning Test

## Discussion

In this study, we have demonstrated that the administration of nicotinamide after ethanol treatment in early postnatal development prevents alcohol-induced hyperactivity and memory impairment in adult mice. The memory impairment in contextual fear conditioning and behavioral hyperactivity both correlated with an increase in neuronal death in cingulate cortex and hippocampus. Administration of nicotinamide protected against this ethanol-induced apoptosis, suggesting a link between neuronal loss during brain development and behavioral disturbances observed in the adult mice. Interestingly, we established that administration of ethanol in postnatal mice induced extensive neuronal death predominantly in the ventral region of the hippocampus. This is consistent with recently published findings that damage to the ventral part of hippocampus significantly impairs performance in contextual fear conditioning [ 43]. Our findings of an increase in the number of entries into the open arm and in the time of permanence in the open arm in ethanol-treated mice are consistent with data previously published [ 33, 44]. However, to better understand whether this behavioral phenotype is due to an anxiolytic effect or simply to an increase of activity, additional studies are needed.

Neuroprotection by nicotinamide in ethanol toxicity appears to function at the mitochondrial level. In the present study, we show that nicotinamide in vivo prevents ethanol-induced cytochrome-c release and the following caspase-3 activation. Our data are consistent with previous studies showing that nicotinamide inhibits oxygen-glucose deprivation-induced neuronal death in vitro, by preventing release of cytochrome-c [ 23]. Although the determination of the precise mechanisms underlying the effects of nicotinamide require furthers analysis, it is possible that nicotinamide may function in stabilizing the cellular energy metabolism [ 16]. During an oxidative stress, depletion of NAD+ is considered a critical factor in precipitating cell death due to compromised energy supply [ 45]. Administration of nicotinamide increases the amount of NAD+ in the brain, preventing its depletion and consequent energetic decline [ 17, 46]. This is consistent with a recent study in which increasing NAD+ biosynthesis prevented axonal degeneration [ 47].

Although there are other studies showing a possible protective effect of antioxidants in preventing ethanol-induced apoptotic neuronal death, to our knowledge this is the first treatment that has been shown to work at the molecular, cellular, and behavioral levels. It has been shown that treatment with the synthetic antioxidant ebselen decreases ethanol-induced apoptotic cell death of young neurons in the adult dentate gyrus [ 48]. Administration of an active fragment of the glial-derived activity-dependent neuroprotective protein, which has potent anti-oxidative properties, prevents ethanol-induced growth retardation in mouse whole-embryo culture and neurotoxicity in vitro [ 49]. Other studies have demonstrated that vitamin E and β-carotene protect against ethanol-induced neuronal death in vitro [ 50, 51]. However, the protective effects of vitamin E in vivo are controversial. While Heaton et al. [ 52] have shown that vitamin E protects against ethanol-induced loss of Purkinje cells of the cerebellum, Tran et al. [ 53] have found that vitamin E does not prevent ethanol-induced caspase-3 activation, cerebellar damage, or behavioral impairment. Furthermore, it has been shown that while a pretreatment with a large dose of vitamin E prevents ethanol-induced cell loss and oxidative stress in the neonatal rat hippocampus, this treatment did not prevent behavioral impairment in ethanol-exposed mice [ 54].

Although nicotinamide was previously used in another model of FAS [ 55], the present study has demonstrated for the first time that nicotinamide exerts a protective effect against ethanol-induced apoptotic cell death and adult neurobehavioral disturbances. We tested nicotinamide between 0 h and 8 h after administration of ethanol, and while we saw the highest level of protection between 0 h and 2 h, nicotinamide treatment retained some effect even after a delay of 8 h ( Figure 1E and 1G; Table S4). We used a dose of ethanol that achieved extremely high blood levels that were maintained for more than 12 h, and yet we were able to reverse the neurobiological effects of ethanol. An important aspect of our study is that nicotinamide has been used for several years in some clinical trials to treat type-I diabetes and bullous pemphigoid (a chronic, autoimmune, blistering disease), orally and in large daily doses for up to five years with little or no side effects [ 56– 59]. Furthermore, there is no evidence that nicotinamide is teratogenic by itself [ 60]. In conclusion, although additional studies are necessary, we suggest, based on the data presented here, that nicotinamide, a readily available and safe agent, could eventually be used in the treatment and prevention of the devastating sequellae of FAS.

## Supporting Information

Alternative Language Abstract S1 Italian Translation of the Abstract (29 KB PDF).

Alternative Language Abstract S2 Spanish Translation of the Abstract (35 KB PDF).

Figure S1 Ethanol Induces Activation of Caspase-3 (A) Ethanol was injected into P7 mice, and activation of caspase-3 was detected at a different time point by Western blot. (B and C) 1-methylnicotinamide does not prevent ethanol-induced activation of caspase-3. P7 mice were injected with ethanol and nicotinamide or 1-methylnicotinamide, an analogue of nicotinamide, administered 2 h after ethanol exposure. At the dose of 1 mg/g, 1-methyl-nicotinamide was toxic. Western blot analysis of activated caspase-3 12 h after ethanol exposure (B) and correspondent densitometric quantification (ANOVA *F _4,18_* = 10.705 *p* = 0.0014; *n* = 4–5 each treatment group) (C).
Values are shown as mean ± SD. Multiple comparison by Bonferroni's procedure revealed a significant difference between the ethanol treatment group and all other groups. No statistical differences were present between ethanol and ethanol plus 1-methyl-nicotinamide.
(1.1 MB PDF).

Table S1 Experimental Groups (53 KB DOC).

Table S2 Nicotinamide Inhibits Ethanol-Induced Caspase-3 Activation Western blot densitometric analysis of caspase-3 activation 12 h after ethanol treatment and different doses of nicotinamide administration.
(31 KB DOC).

Table S3 Nicotinamide Inhibits Ethanol-Induced Caspase-3 Activation Western blot densitometric analysis of caspase-3 activation 24 h after ethanol treatment.
(28 KB DOC).

Table S4 Nicotinamide Inhibits Ethanol-Induced Caspase-3 Activation Western blot densitometric analysis of caspase-3 activation 12 h after ethanol treatment and different time points of nicotinamide administration.
(31 KB DOC).

Table S5 Nicotinamide Inhibits Ethanol-Induced Caspase-3 Activation Western blot densitometric analysis of caspase-3 activation 12 h after different doses of ethanol administration.
(31 KB DOC).

Table S6 Nicotinamide Inhibits Ethanol-Induced Caspase-3 Activation Western blot densitometric analysis of caspase-3 activation 12 h after ethanol treatment using nicotinamide or an analogue of nicotinamide.
(29 KB DOC).

Table S7 Nicotinamide Blocks Release of Citochrome-c from Mitochondria Western blot densitometric analysis of cytochrome-c release from mitochondria 12 h after ethanol administration.
(28 KB DOC).

**Patient Summary**

**Background**

When a woman drinks alcohol while she is pregnant, the alcohol goes to the baby through her bloodstream and can harm the baby. Some babies whose mothers had been drinking alcohol during pregnancy are born with fetal alcohol syndrome (FAS), a serious condition that can affect a child throughout life. Children with FAS have problems with development, learning, behavior, and social skills. In many Western countries, FAS is a common cause of mental retardation. Nicotinamide is a drug that has been used orally in patients with type-I diabetes and bullous pemphigoid (a chronic, autoimmune, blistering disease) and is generally well-tolerated.

**Why Was This Study Done?**

Nicotinamide has also been found to have neuroprotective properties (that means it can protect nerve cells). Because some of the problems associated with FAS are thought to be caused by the alcohol killing cells in the baby's brain, the researchers wanted to test whether nicotinamide could prevent some of the consequences of alcohol exposure. Before one can start testing such potential treatments in humans, it is necessary to do extensive studies in animal models (and only a small proportion of ideas that look promising in animal studies will eventually turn into useful treatments for human patients).

**What Did the Researchers Do and Find?**

They injected mouse pups shortly after birth with alcohol. Mouse brain development happens a bit later than in humans, and the time shortly after birth in mice corresponds to brain development in human babies during the third trimester of pregnancy. They found that this single exposure to alcohol (which is comparable with one-time excessive drinking in a pregnant mother) caused the death of brain cells in the mouse pups as well as a range of behavioral abnormalities after the pups had grown into adult mice. When they followed the injection of alcohol with a second injection of nicotinamide two hours later, the number of brain cells that died was no greater than during normal brain development, and the mice did not show behavioral abnormalities as adults.

**What Do These Findings Mean?**

This means that it might be possible, some years from now, to prevent some of the alcohol damage to the baby if the mother gets nicotinamide treatment soon after she was drinking alcohol. However, these are early-stage experiments done in a mouse model of FAS, and much more work is needed before it will become clear whether this or a similar treatment would work at all in humans. Moreover, FAS causes severe birth defects that are entirely preventable if a pregnant woman doesn't drink. Changing the behavior of pregnant women (and those who might become pregnant) therefore must be and will remain the focus of medical care.

**Where Can I Get More Information Online?**

The following Web sites have information on fetal alcohol syndrome.

National Organization of Fetal Alcohol Syndrome:

http://www.nofas.org/about

The US Centers for Disease Control and Prevention (a list of relevant publications):

http://www.cdc.gov/ncbddd/fas/faspub.htm

Australian Health Insite:

http://www.healthinsite.gov.au/topics/Foetal_Alcohol_Syndrome

Health on the Net Foundation:

http://www.hon.ch/Dossier/MotherChild/neonatal_problems/fetal_alcohol.html

US National Institute on Alcohol Abuse and Alcoholism:

http://www.niaaa.nih.gov/AboutNIAAA/Interagency/AboutFAS.htm

## Abbreviations

ADN: antero dorsal nucleus
AVN: antero ventral nucleus
CX: cortex
DG: dental gyrus
EtOH: ethanol
FAE: fetal alcohol effect
FAS: fetal alcohol syndrome
LDN: lateral dorsal nucleus
NAD+: β-nicotinamide adenine ninucleotide
NeuN: neuronal nuclear protein
Nic: nicotinamide
P[number]: postnatal day [number]
SD: standard deviation
