# Supplementary material for: Nicotinamide Protects against Ethanol-Induced Apoptotic Neurodegeneration in the Developing Mouse Brain
Source: PLoS Med. 2006 Feb 21;3(4):e101. doi: 10.1371/journal.pmed.0030101 (PMC1370925; doi:10.1371/journal.pmed.0030101)
Supplement: Supplementary file 1 [file pmed.0030101.sd001.pdf]

Translation of the abstract (Nicotinamide Protects Against Ethanol-Induced Apoptotic Neurodegeneration in the Developing Brain) into Italian by Alessandro Ieraci.

Introduzione

L'esposizione all'alcool durante lo sviluppo del cervello puo' causare una sindrome neurologica conosciuta come sindrome fetale alcolica (FAS). L'etanolo induce la morte apoptotica dei neuroni immaturi particolarmente durante il periodo di sviluppo definito sinaptogenesi.

La somministrazione di una singola dose di etanolo in topi di sette giorni promuove l'attivazione della caspasi-3 e una estesa morte apoptotica dei neuroni nel telencefalo, fornendo una possible spiegazione della microcefalia osservarta nei soggetti affetti da FAS. Il presente studio e' stato condotto per determinare se la nicotinamide puo' prevenire la neurodegenerazione indotta dall'alcol.

Metodi e risultati

Topi di 7 giorni sono stati trattati con nicotinamide, in un intervallo di tempo compreso tra le 0 e le 8 ore, dopo somministrazione di una singola dose di etanolo (5 g/kg). Gli effetti della nicotinamide nell'attivazione della caspasi-3 e nel rilascio del citocromo-C promossi dall'etanolo sono stati analizzati tramite western blot (n=4-7/gruppo). La densita' del numero di cellule positive alla colorazione per il Fluoro-Jade B e il NeuN e' stata conteggiata nella corteccia cingulata, nella regione CA1 dell'ippocampo e nel nucleo dorso-laterale del talamo (n=5-6/gruppo). Per studiare il comportamento dei topi adulti si e' fatto uso dell'arena aperta (open field), del labirinto a croce rialzato (elevated plus maze), e del test di paura condizionata (fear conditioning test) (n=31-34/gruppo). La sommministrazione di nicotinamide riduce l'attivazione della caspasi-3 (85.14±4.1%) ed il rilascio del citocromo-C (80.78±4.39%), promossi dall'etanolo, nel telencefalo di topi in eta' postnatale. Inoltre la somministarzione di nicotinamide riduce la morte apoptotica dei neuroni indotta dall'etanolo. Topi trattati con l'etanolo dimostrano una ridotta risposta nel fear conditioning test e una maggiore attivita' nell'open field e nell'elevated

plus maze. La somministrazione di nicotinamide, nei topi trattati con l'etanolo, previene tutti i disturbi comportamentali sopracitati.

Conclusioni

L'insieme dei nostri dati indicano che la nicotinamide puo' prevenire gli effetti deleteri promossi dall'etanolo nel cervello durante lo sviluppo. Questi risultati suggeriscono di conseguenza che la nicotinamide potrebbe essere un valido trattamento nella terapia preventiva della sindrome fetale alcolica.
