# Supplementary material for: Nicotinamide Protects against Ethanol-Induced Apoptotic Neurodegeneration in the Developing Mouse Brain
Source: PLoS Med. 2006 Feb 21;3(4):e101. doi: 10.1371/journal.pmed.0030101 (PMC1370925; doi:10.1371/journal.pmed.0030101)
Supplement: Supplementary file 2 [file pmed.0030101.sd002.pdf]

Translation of the abstract (Nicotinamide Protects Against Ethanol-induced Apoptotic Neurodegeneration in the Developing Brain) into Spanish by Daniel G. Herrera.

Introducción

La exposición del cerebro en desarrollo al alcohol puede causar un síndrome neurológico conocido como síndrome alcohólico fetal. El alcohol induce la muerte neuronal por apoptosis en etapas especificas del desarrollo cerebral, particularmente en una etapa conocida como "crecimiento cerebral agudo" ("brain growth spurt" en ingles). La administración de una única dosis de etanol en ratones de siete días de edad inicia una cascada de eventos que incluyen la activación de caspasa-3 y muerte neuronal masiva en el telencéfalo que podría explicar la microencefalia que es parte del síndrome alcohólico fetal en humanos. El presente estudio intenta determinar si la nicotinamida podría prevenir la neurodegeneración inducida por el alcohol.

Métodos y Hallazgos

Ratones de siete días de edad recibieron una única dosis de etanol (5g/kg) y posteriormente se administro nicotinamida desde 0 a 8 horas *después* de la dosis de etanol. Los efectos de la nicotinamida sobre los efectos del alcohol en la activación de la caspasa-3 y la liberación de citocromo C desde las mitocondrias fueron estudiados con Western blot (n= 4-7/grupo.) La densidad de células positivas a FluoroJade B o a NeuN fue determinada en la corteza cingulada, la región CA1 del hipocampo y en el núcleo laterodorsal del tálamo (n= 5-6/grupo.) Tests de conducta de campo abierto (open field), laberinto en cruz elevada (plus maze) y condicionamiento por miedo (fear conditioning), se hicieron en los ratones una vez alcanzaron la edad adulta (n=31-34/ grupo.) La nicotinamida redujo la activación de caspasa-3 (85.14±4.1%) y la liberación de citocromo C (80.78 ± 4.39) en el telencéfalo postnatal. La nicotinamida previno la muerte neuronal apoptotica inducida por el etanol. Demostramos que los ratones expuestos a etanol tenían dificultades de aprendizaje en el test de condicionamiento y un aumento en la actividad motora inespecífica en el

campo abierto y en el laberinto. La administración de nicotinamida en animales expuestos al alcohol impidió que estos trastornos conductuales ocurrieran.

Conclusión

Nuestros hallazgos indican que la nicotinamida podría inhibir los efectos deletéreos del alcohol en el cerebro en desarrollo. Estos resultados sugieren que la nicotinamida puede ser prometedora como una terapia preventiva en el tratamiento del síndrome alcohólico fetal.
