# Supplementary material for: Nicotinamide Protects against Ethanol-Induced Apoptotic Neurodegeneration in the Developing Mouse Brain
Source: PLoS Med. 2006 Feb 21;3(4):e101. doi: 10.1371/journal.pmed.0030101 (PMC1370925; doi:10.1371/journal.pmed.0030101)
Supplement: Supplementary file 4 [file pmed.0030101.st001.doc]

**Table S1.**

**Experimental groups.**

| **Experiment** | | **n** | **Age of treatment** | **Weight (g)** | **EtOH (g/kg)** | **Nic (mg/g)** | **Time**  **Nic** ^a^ **(h)** | **Time Sacrificed** **^a^** | **Age of Test** | **Results described** **^b^** |
| --- | --- | --- | --- | --- | --- | --- | --- | --- | --- | --- |
|  |  |  |  |  |  |  |  |  |  |  |
| **Western** | |  |  |  |  |  |  |  |  |  |
| ***Caspase-3*** | |  |  |  |  |  |  |  |  |  |
|  | Doses of Nic | 5-6 | P7 | 3.5-5.5 | 5 | 0.25; 0.5; 1 | 2 | 12 h | NA | Fig. 1 A, C, table S2 |
|  | 24hrs | 6 | P7 | 3.5-5.5 | 5 | 1 | 2 | 24 h | NA | Fig. 1 B, D, table S3 |
|  | Different time point of Nic injection. | 6-7 | P7 | 3.5-5.5 | 5 | 1 | 0; 2; 4; 8 | 12 h | NA | Fig. 1 E, G, table S4 |
|  | Doses of EtOH | 5 | P7 | 3.5-5.5 | 2.5; 4; 5 | 1 | 2 | 12 h | NA | Fig. 1 F, H, table S5 |
|  | Analogue of Nic | 4-5 | P7 | 3.5-5.5 | 5 | 0.5 | 2 | 12 h | NA | Fig. S1 B, C, table S6 |
| ***Cytochrome-C*** | | 6 | P7 | 3.5-5.5 | 5 | 1 | 2 | 12 h | NA | Fig. 2 A, C, table S7 |
|  |  |  |  |  |  |  |  |  |  |  |
| **Ethanol level quantification** | | |  |  |  |  |  |  |  |  |
|  |  | 4 | P7 | 3.5-5.5 | 5 | 1 | 2 | 4; 8; 12; 24h | NA | Fig. 2 B, D |
|  |  |  |  |  |  |  |  |  |  |  |
| **Morphological Analysis** | | |  |  |  |  |  |  |  |  |
|  | ***Fluoro-Jade B Staining*** | 5 6 | P7 | 3.5-5.5 | 5 | 1 | 2 | 24 h | NA | Fig. 3 A, B |
|  | ***NeuN IHC*** | 5 | P7 | 3.5-5.5 | 5 | 1 | 2 | 14 days | NA | Fig. 3 C |
|  |  |  |  |  |  |  |  |  |  |  |
| **Behavioral Test** | |  |  |  |  |  |  |  |  |  |
|  | ***Open Field Test*** | 31-34 | P7 | 3.5-5.5 | 5 | 1 | 2 | NA | 8 weeks | Table 1 |
|  | ***Plus Maze*** | 31-34 | P7 | 3.5-5.5 | 5 | 1 | 2 | NA | 8 weeks | Table 1 |
|  | ***Fear Conditioning Test*** | 31-34 | P7 | 3.5-5.5 | 5 | 1 | 2 | NA | 8 weeks | Table 2 |
|  |  |  |  |  |  |  |  |  |  |  |
|  |  |  |  |  |  |  |  |  |  |  |

^a^ After ethanol treatment

^b^ Experiments are described in the methods section and the results are show in the figures and tables indicated in this column.

EtOH=Ethanol; Nic=Nicotinamide; IHC=ImmunoHistoChemistry; NA=not available.
