# Supplementary material for: Nicotinamide Protects against Ethanol-Induced Apoptotic Neurodegeneration in the Developing Mouse Brain
Source: PLoS Med. 2006 Feb 21;3(4):e101. doi: 10.1371/journal.pmed.0030101 (PMC1370925; doi:10.1371/journal.pmed.0030101)
Supplement: Supplementary file 5 [file pmed.0030101.st002.doc]

# Table S2.

Western blot densitometric analysis of caspase-3 activation 12 hours after ethanol treatment and different doses of nicotinamide administration.

|  |  |  |  | ***P*-value** **^b^** | |
| --- | --- | --- | --- | --- | --- |
| Treatment | **n** | **Mean** **^a^** | **SD** | **Saline** | **EtOH** |
| Saline | 6 | 100.20 | 0.35 | 0.0000 | <0.0001* |
| EtOH | 6 | 1949.15 | 925.79 | <0.0001* | 0.00 |
| EtOH + Nic (0.25mg/g) | 5 | 895.47 | 715.19 | 0.0163 | 0.0021* |
| EtOH + Nic (0.5mg/g) | 5 | 778.66 | 353.77 | 0.0300 | 0.0005* |
| EtOH + Nic (1mg/g) | 6 | 269.11 | 188.12 | 0.5909 | <0.0001* |
| Saline+ Nic (1mg/g) | 5 | 105.02 | 22.37 | 0.9877 | <0.0001* |
|  |  |  |  |  |  |

^a^ % of control.

^b^ P-value from ANOVA following by Bonferroni’s procedure. Comparisons are considered significant with a *P*-value less than 0.033.

EtOH=Ethanol; Nic=Nicotinamide.
