# Supplementary material for: Nicotinamide Protects against Ethanol-Induced Apoptotic Neurodegeneration in the Developing Mouse Brain
Source: PLoS Med. 2006 Feb 21;3(4):e101. doi: 10.1371/journal.pmed.0030101 (PMC1370925; doi:10.1371/journal.pmed.0030101)
Supplement: Supplementary file 6 [file pmed.0030101.st003.doc]

# Table S3.

# Western blot densitometric analysis of caspase-3 activation 24 hours after ethanol treatment.

|  |  |  |  | ***P*-value**^b^ | |
| --- | --- | --- | --- | --- | --- |
| Treatment | **n** | **Mean**^a^ | **SD** | **Saline** | **EtOH** |
| Saline | 6 | 100.516 | 0.873 | 0 | 0.0002* |
| EtOH | 6 | 299.327 | 120.008 | 0.0002* | 0 |
| EtOH + Nic | 6 | 161.678 | 75.118 | 0.1756 | 0.0053* |
| Saline + Nic | 6 | 98.933 | 4.752 | 0.9743 | 0.0006* |
|  |  |  |  |  |  |

^a^ % of control.

^b^ P-value from ANOVA following by Bonferroni’s procedure. Comparisons are considered significant with a *P*-value less than 0.083.

EtOH=Ethanol; Nic=Nicotinamide.
