# Supplementary material for: Nicotinamide Protects against Ethanol-Induced Apoptotic Neurodegeneration in the Developing Mouse Brain
Source: PLoS Med. 2006 Feb 21;3(4):e101. doi: 10.1371/journal.pmed.0030101 (PMC1370925; doi:10.1371/journal.pmed.0030101)
Supplement: Supplementary file 7 [file pmed.0030101.st004.doc]

# Table S4.

# Western blot densitometric analysis of caspase-3 activation 12 hours after ethanol treatment and different time points of nicotinamide administration.

|  |  |  |  | ***P*-value** **^b^** | |
| --- | --- | --- | --- | --- | --- |
| Treatment | **n** | **Mean** **^a^** | **SD** | **Saline** | **EtOH** |
| Saline | 7 | 100.011 | 0.116 | 0 | <0.0001* |
| EtOH | 7 | 2205.991 | 471.157 | <0.0001* | 0 |
| EtOH +Nic (0h) | 6 | 748.972 | 475.276 | 0.0228 | <0.0001* |
| EtOH +Nic (2h) | 7 | 608.165 | 324.554 | 0.0602 | <0.0001* |
| EtOH +Nic (4h) | 6 | 818.449 | 788.864 | 0.0125 | <0.0001* |
| EtOH +Nic (8h) | 6 | 1296.041 | 578.3225 | 0.0001* | 0.0020* |
|  |  |  |  |  |  |

^a^ % of control.

^b^ P-value from ANOVA following by Bonferroni’s procedure. Comparisons are considered significant with a *P*-value less than 0.033.

EtOH=Ethanol; Nic=Nicotinamide.
