# Supplementary material for: Nicotinamide Protects against Ethanol-Induced Apoptotic Neurodegeneration in the Developing Mouse Brain
Source: PLoS Med. 2006 Feb 21;3(4):e101. doi: 10.1371/journal.pmed.0030101 (PMC1370925; doi:10.1371/journal.pmed.0030101)
Supplement: Supplementary file 8 [file pmed.0030101.st005.doc]

# Table S5.

# Western blot densitometric analysis of caspase-3 activation 12 hours after different doses of ethanol administration.

|  |  |  |  | ***P*-value** **^b^** | | | |
| --- | --- | --- | --- | --- | --- | --- | --- |
| Treatment | **n** | **Mean** **^a^** | **SD** | **Saline** | **EtOH (2.5g/kg)** | **EtOH (4g/kg)** | **EtOH (5g/kg)** |
| Saline | 5 | 100.023 | 0.074 | 0 | 0.9406 | <0.0001* | <0.0001* |
| EtOH (2.5g/kg) | 5 | 117.024 | 28.293 | 0.9406 | 0 | <0.0001* | <0.0001* |
| EtOH (2.5g/kg)+Nic | 5 | 98.862 | 31.157 | 0.9959 | 0.9365 | <0.0001* | <0.0001* |
| EtOH (4g/kg) | 5 | 1305.870 | 836.994 | <0.0001* | <0.0001* | 0 | 0.029 |
| EtOH (4g/kg)+Nic | 5 | 397.821 | 97.420 | 0.1984 | 0.2245 | 0.0004* | <0.0001* |
| EtOH (5g/kg) | 5 | 1826.182 | 359.432 | <0.0001* | <0.0001* | 0.029 | 0 |
| EtOH (5g/kg)+Nic | 5 | 486.633 | 230.677 | 0.0983 | 0.1132 | 0.0011* | <0.0001* |
|  |  |  |  |  |  |  |  |

^a^ % of control.

^b^ P-value from ANOVA following by Bonferroni’s procedure. Comparisons are considered significant with a *P*-value less than 0.024.

EtOH=Ethanol; Nic=Nicotinamide.
