# Supplementary material for: Nicotinamide Protects against Ethanol-Induced Apoptotic Neurodegeneration in the Developing Mouse Brain
Source: PLoS Med. 2006 Feb 21;3(4):e101. doi: 10.1371/journal.pmed.0030101 (PMC1370925; doi:10.1371/journal.pmed.0030101)
Supplement: Supplementary file 9 [file pmed.0030101.st006.doc]

# Table S6.

# Western blot densitometric analysis of caspase-3 activation 12 hours after ethanol treatment using nicotinamide or an analogue of nicotinamide.

|  |  |  |  | ***P*-value** **^b^** | |
| --- | --- | --- | --- | --- | --- |
| Treatment | **n** | **Mean** **^a^** | **SD** | **Saline** | **EtOH** |
| Saline | 5 | 100.319 | 0.366 | 0 | <0.0001* |
| EtOH | 5 | 1969.611 | 906.797 | <0.0001* | 0 |
| EtOH + Nic | 5 | 774.680 | 395.373 | 0.0873 | 0.0049* |
| EtOH + 1-Met-Nic | 4 | 1839.417 | 883.579 | 0.0003* | 0.7459 |
| Saline + 1-Met-Nic | 4 | 118.021 | 35.186 | 0.9648 | 0.0002* |
|  |  |  |  |  |  |

^a^ % of control.

^b^ P-value from ANOVA following by Bonferroni’s procedure. Comparisons are considered significant with a *P*-value less than 0.005. EtOH=Ethanol; Nic=Nicotinamide; 1-Met-Nic =1-Methyl-Nicotinamide.
