# Supplementary material for: Nicotinamide Protects against Ethanol-Induced Apoptotic Neurodegeneration in the Developing Mouse Brain
Source: PLoS Med. 2006 Feb 21;3(4):e101. doi: 10.1371/journal.pmed.0030101 (PMC1370925; doi:10.1371/journal.pmed.0030101)
Supplement: Supplementary file 10 [file pmed.0030101.st007.doc]

# Table S7.

# Western blot densitometric analysis of cytochrome-C release from mitochondria 12 hours after ethanol administration.

|  |  |  |  | ***P*-value** **^b^** | |
| --- | --- | --- | --- | --- | --- |
| **Treatment** | **n** | **Mean** **^a^** | **SD** | **Saline** | **EtOH** |
| Saline | 6 | 100.368 | 0.207 | 0 | <0.0001* |
| EtOH | 6 | 2164.917 | 694.503 | <0.0001* | 0 |
| EtOH +Nic | 6 | 416.417 | 232.710 | 0.1511 | <0.0001* |
| Saline+Nic | 6 | 114.767 | 38.905 | 0.9465 | <0.0001* |
|  |  |  |  |  |  |

^a^ % of control.

^b^ P-value from ANOVA following by Bonferroni’s procedure. Comparisons are considered significant with a *P*-value less than 0.083.

EtOH=Ethanol; Nic=Nicotinamide.
